# Predictive factors for insertion torque values in transalveolar sinus lift procedures

**DOI:** 10.1038/s41405-025-00297-w

**Published:** 2025-03-03

**Authors:** Ziad Albash, Ali Khalil

**Affiliations:** 1 MSc in Oral and Maxillofacial surgery, Oral and Maxillofacial surgery department, Faculty of Dentistry, Tishreen University, Lattakia, Syria; 2 Sakan aliatiba’i, almashrue alsaabieu, Lattakia, Lattakia government Syria; 3 Associate Professor, Oral and Maxillofacial surgery department, Faculty of Dentistry, Manara University, Lattakia, Syria

**Keywords:** Dentistry, Dental implants

## Abstract

**Objectives:**

The objective of this retrospective study was to investigate the impact of bone-related factors such as initial bone height (IBH), imaginary pixel unit (IPU), bone diameter (BD), crestal cortical thickness (CCT), and sinus floor thickness (SFT) in transalveolar sinus lift procedures using threaded bone expanders without bone graft materials.

**Materials and methods:**

This retrospective cohort study was carried out on patients who had reported to the department of oral and maxillofacial surgery at Tishreen University between January 2018 and June 2024. The sample consisted of patients who had transalveolar sinus lift using motorized threaded bone expanders with simultaneous hybrid dental implants placement without bone graft materials. The bone-related factors, including initial bone height (IBH), imaginary pixel unit (IPU), bone diameter (BD), crestal cortical thickness (CCT), and sinus floor thickness (SFT) were analyzed using CBCT scans that were taken preoperatively. Statistical analysis was performed to assess the relationship of the factors and insertion torque of the dental implant. The statistical analysis includes descriptive statistics for all variables mentioned. A Uni-variate linear regression (Spearman’s correlation) and multivariate linear regression were performed to assess the relation between Insertion torque and the explanatory variables. The *p* values < 0.05 were considered to be statistically significant. The Newey West correction for heteroskedasticity was applied.

**Results:**

In this retrospective study, 124 hybrid implants (cylindrical in coronal section and conical in apical section) were placed in 113 patients (53 males and 60 females) in the posterior region of the maxilla in conjunction with transalveolar sinus lift. In the Uni-variate analysis, we observed a strong positive correlation between IT and IPU, a weak positive correlation between IT and SFT, and between IT and CCT. There was no correlation found between IT and IBH, as well as between IT and BD. Through multivariate analysis, we found that IPU and CCT were associated with higher values of IT, whereas IBH, BD, and SFT were not significantly associated with the value of insertion torque.

**Conclusions:**

Our study indicates that, within its limitations, insertion torque values in transalveolar sinus lift procedures using threaded bone expanders and hybrid implants are primarily influenced by cortical bone thickness and imaginary pixel unit, while factors such as sinus floor thickness, initial bone height, and bone diameter do not significantly affect these values.

## Introduction

The rehabilitation of the posterior maxillary region poses a significant challenge for dental implant practitioners. This difficulty primarily stems from the insufficient bone density and height in this area, which hinders the placement of dental implants capable of withstanding occlusal stresses [1, 2]. The posterior maxilla includes the upper jaw area extending from the first premolar to the pterygoid process and is a fusion of several bones, such as the maxillary bone, palatine bone, zygomatic bone, and pterygoid plates of the sphenoid bone. The maxillary sinus floor serves as the cranial border, while the crestal bone acts as the caudal border [3].

Primary stability pertains to the biomechanical stability of an implant immediately following its placement into the prepared site [4]. This stability is established through the mechanical contact between the implant and the surrounding bone [5]. Any movement of the implant exceeding 50–100 microns has the potential to affect the osseointegration process, potentially leading to fibrous integration and eventual implant failure [4]. Hence, an implant is considered stable when its movement remains below this specified threshold [6].

The primary stability of dental implants is influenced by various factors, such as bone-related factors [7, 8], implant-related factors [9–11], and surgical technique [12]. These factors play a crucial role in predicting the primary stability of dental implants when they are fully surrounded by bone. In the context of transalveolar sinus lift, the apical part of the dental implant is not encompassed by living bone, which is known as the implant protrusion length (IPL) [13, 14]. Hence, the findings from prior studies regarding the factors influencing the primary stability of dental implants may not directly apply to transalveolar sinus lift procedures.

The assessment techniques for primary stability of implants can be classified into invasive methods that have an impact on the osseointegration process and non-invasive methods that do not disrupt it [15]. Key non-invasive methods include radiographic analysis, insertion torque measurement, and resonance frequency analysis (RFA) [15]. On the other hand, recording removal torque values and histological analysis are regarded as significant complementary methods for evaluating primary stability [15].

Insertion torque is a commonly used measure of primary stability in clinical studies and daily practice. It quantifies the energy needed to cut a unit volume of bone during implant insertion (expressed in mJ/mm^3^) [16]. Typically, insertion torque values are converted to units of Newton.cm (Ncm) from their base units by applying the appropriate conversion relationship [16]. The insertion torque of the implant can be measured manually using an insertion arm during the implant placement process, or it can be automatically determined by the surgical drilling device as the implant is lowered into position [17]. While this method is regarded as one of the simpler ways to evaluate implant stability, it has limitations. It can only be utilized at the time of implant insertion, lacking the ability to track stability changes during the healing phase. Additionally, it relies on the cortical bone quantity and does not provide an accurate representation of the percentage of bone contact with the implant [18, 19].

Transalveolar sinus floor elevation is a reliable and predictable technique for rehabilitating the posterior region with dental implants [1, 13]. The bone density deficient nature of this area necessitates methods that enhance bone density to attain adequate primary stability for successful implant placement. The osteotome-based technique introduced by Summers [20] and its modifications [21–23] represent the prevailing approach to transalveolar sinus floor elevation. The osteotome not only displaces the cortical sinus floor apically but also increases bone density both apically and laterally [20]. Other techniques, such as osseodensification [24], bioactive kinetic screw (BKS) [25, 26], and threaded bone expanders [27], have been suggested as alternatives to the osteotome technique, utilizing rotating burs instead of a hammer.

The objective of this study was to identify predictive factors for insertion torque values in transalveolar sinus lift procedures. The specific goals of the study were to investigate the impact of bone-related factors including, initial bone height (IBH), imaginary pixel unit (IPU), bone diameter (BD), crestal cortical thickness (CCT), and sinus floor thickness (SFT) in transalveolar sinus lift procedures using threaded bone expanders without bone graft materials.

## Materials and methods

### Ethical approval and consent

This retrospective study was conducted in accordance with the Declaration of Helsinki for human studies and was approved by the Ethics Committee of Tishreen University (Ethical Permission No. 707 on 17-12-2019). The patients were informed about the details of the surgery, and all of the subjects gave their written informed consent for inclusion prior to the study.

### Study design

This study was conducted on patients who presented to the department of oral and maxillofacial surgery at Tishreen University between January 2018 and June 2024. Patient files were carefully reviewed to identify individuals who met the following inclusion criteria.

#### Inclusion criteria

1. Cone Beam Computed Tomography (CBCT) scans available before surgical procedure.
2. Transalveolar sinus lift procedures with simultaneous dental implant placement using threaded bone expanders.
3. Initial bone height (5-8) mm.
4. Patient age between 18-60 years.

#### Exclusion criteria

1. Immediate implant placement procedures.
2. Using of any bone graft or bone substitutes materials.
3. Bone expansion procedures.
4. Previous maxillary sinus surgery.

### Surgical procedure

The transalveolar sinus lift procedure was performed using a specialized sinus lift kit (Sinus Lift Kit; Cowellmedi Inc, South Korea). The kit consists of five conical threaded bone spreaders with different diameters. The tip of the spreaders has a U-shaped blade.

All surgical procedures were carried out by the same surgeon under local anesthesia with 2% lidocaine and 1:100,000 epinephrine (Lidocaine, AVENZOR, Syria). A full-thickness mucoperiosteal flap was raised using a single crestal incision (with or without vertical incisions) to expose the alveolar ridge.

The implant site was first prepared using a 2.2 mm pilot drill, reaching a depth of approximately 1 mm below the sinus floor. The implant bed was then further prepared with a 2.9 twister drill. The transalveolar sinus lift was started with a 3.2 mm spreader at 50 rpm. The extent of lift and the intended implant length were predetermined.

The osteotomy site preparation advanced with larger diameter spreaders until reaching the desired diameter. The implant was then inserted using a motorized handpiece. Each implant was positioned at the crestal bone level (aligning the upper surface of the implant’s shoulder with the crestal bone). After implant insertion, the flap was closed with simple interrupted sutures using 4-0 silk sutures (SilkoMed; MedSilk GmbH, Germany).

The patients were instructed to take amoxicillin/clavulanate 875/125 mg (Augmentin 1000, Maatouk Pharma, Syria) twice daily for 5 days, potassium diclofenac 50 mg (Flam K, Asia Co, Syria) as needed, and to use an antimicrobial mouthwash (Fresh Mouth, BIOGHAR, Syria) for 7 days. Sutures were removed after 7 days.

### Data collection

The following primary variables were registered: Insertion torque (IT), initial bone height (IBH), imaginary pixel unit (IPU), bone diameter (BD), crestal cortical thickness (CCT), and sinus floor thickness (SFT). Secondary variables such as age, sex, location of the implant.

#### Measurement of insertion torque

All implant bed preparation procedures were carried out using an advanced implant motor (DTE Implanter; Woodpecker; China). The torque was limited to 60 N.cm as the maximum insertion torque value to prevent exceeding it. The implant was inserted with a motorized handpiece. The torque value displayed on the motor screen increased as the implant progressed further into the bone. The insertion torque value on the motor screen was noted when the implant reached the crestal bone level.

#### Radiographic measurements

**Fig. 1:**
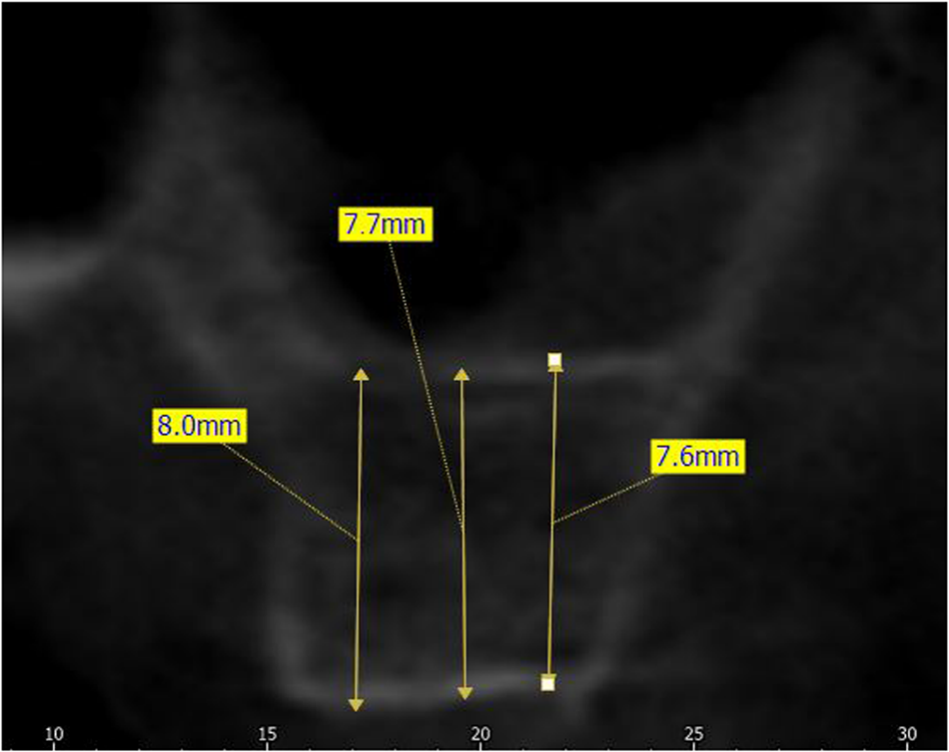
IBH Measurement. Measurement of initial bone height (IBH).

The following variables were assessed using CBCT scan (Carestream Dental CS 9600 LLC, Atlanta, GA, USA) that were taken preoperatively:

**Initial Bone Height (IBH):** was measured parallel to the implant axis as the distance between the alveolar bone crest and sinus floor at the intended implant placement site at three different levels (midpoint, mesial, and distal to the planned implant location) (Fig. 1).

**Fig. 2:**
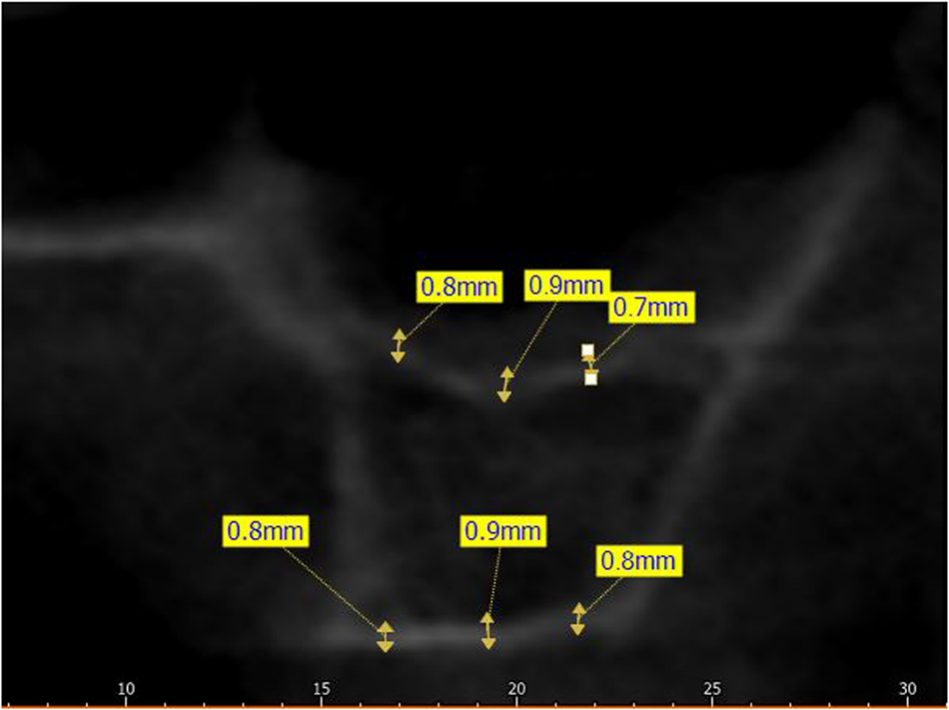
CCT & SFT Measurement. Measurement of crestal cortical thickness (CCT) and sinus floor thickness (SFT).

**Crestal Cortical Thickness (CCT):** was measured as the thickness of the cortical bone at the alveolar bone crest at three different levels (midpoint, mesial, and distal to the planned implant placement site) (Fig. 2).

**Sinus Floor Thickness (SFT):** was measured as the thickness of the cortical bone at the sinus floor at three different levels (midpoint, mesial, and distal to the planned implant placement site) (Fig. 2).

**Fig. 3:**
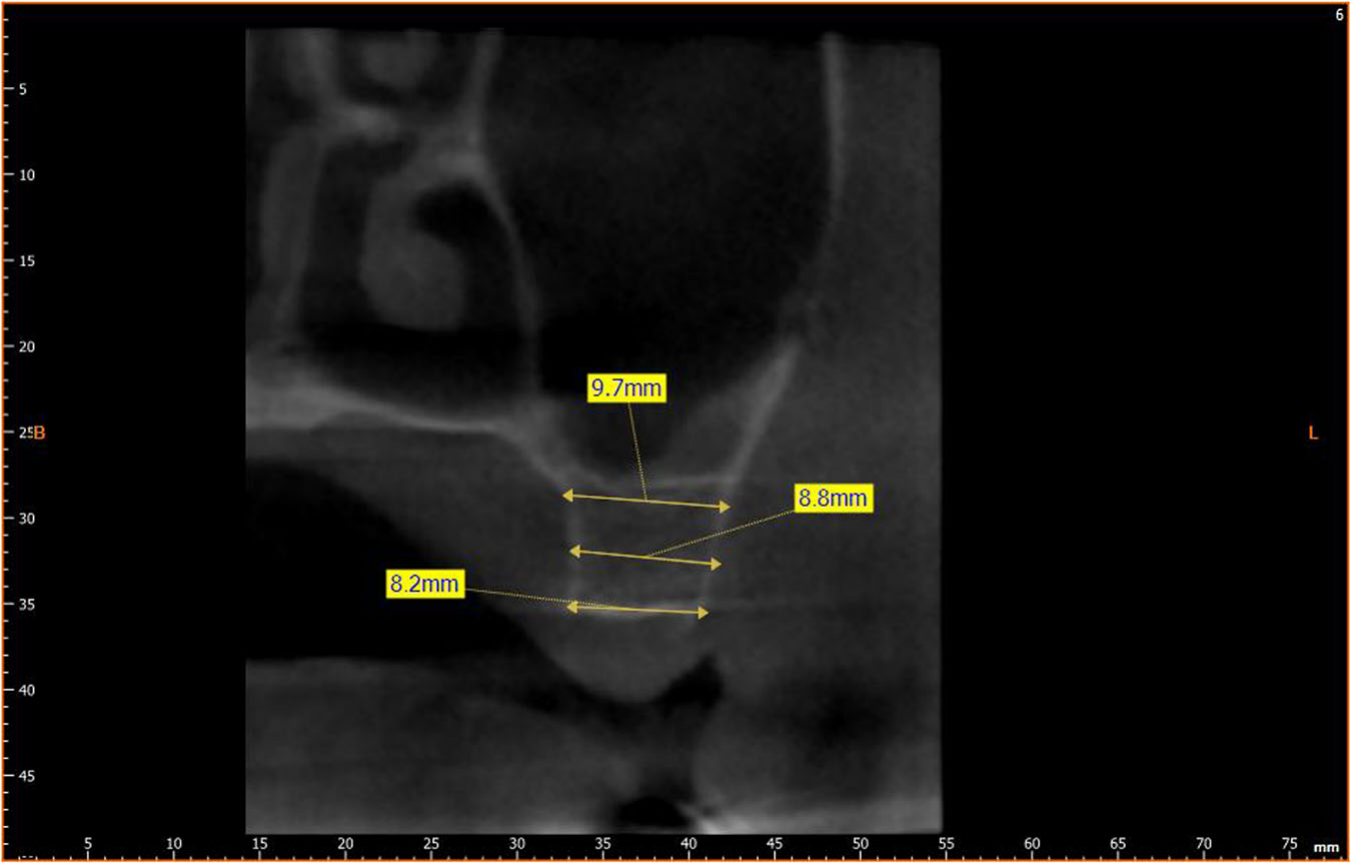
BD Measurement. Measurement of bone diameter (BD).

**Bone Diameter (BD):** was measured perpendicular to the implant axis between the buccal plate and palatal plate at three different levels (Fig. 3). The level of measurements were at distances of 1, 3, and 5 mm from the alveolar crest.

**Fig. 4:**
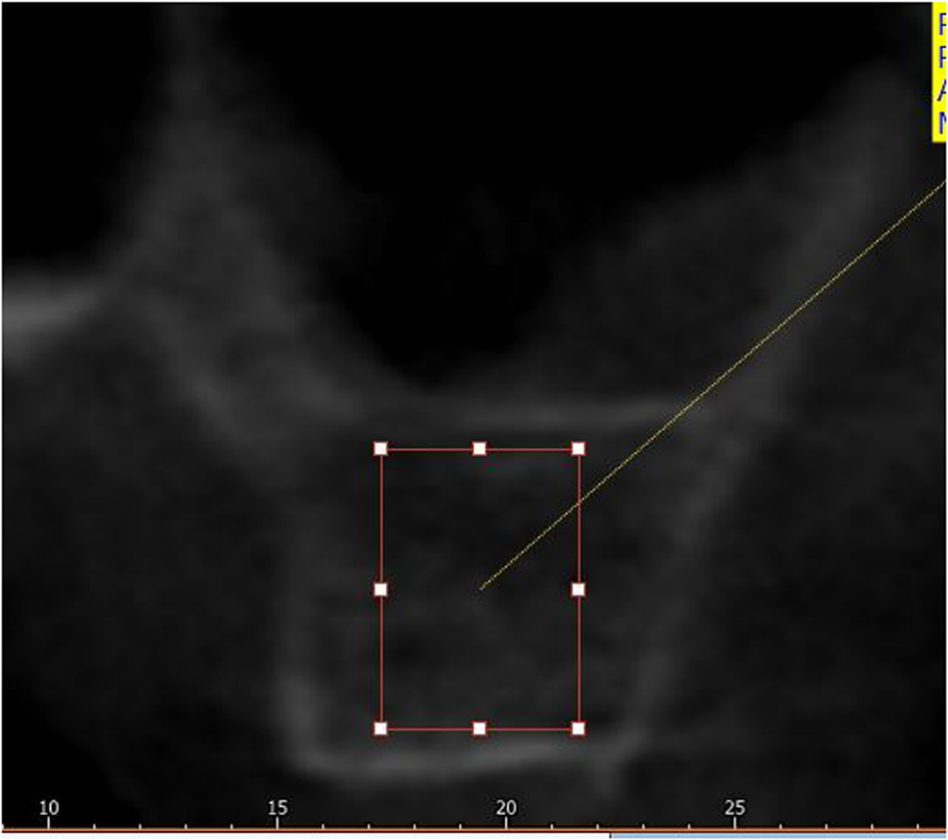
IPU Measurement. Measurement of cancellous imaginary pixel unit (IPU).

**Imaginary pixel unit (IPU):** was measured using the rectangle ROI method at three different sections (midpoint, mesial, and distal to the planned implant placement site) (Fig. 4).

### Interexaminer and Intraexaminer error

The accuracy of measurements was verified prior to assessing the five radiographic variables. In order to assess the reliability of measurements conducted by examiners, the intraclass correlation coefficient (ICC) was employed.

**Interexaminer error**: The radiographic variables from a specific position on CBCT scans were assessed independently by two examiners and subsequently compared.

**Intraexaminer error**: The radiographic variables from a specific position on CBCT scans were evaluated five times by a single examiner, and the resulting measurements were then compared.

To avoid bias, a single surgeon (ZA) conducted all surgical procedures, and radiological assessments were evaluated by an impartial investigator (AK) who was unaware of the patients’ identities.

### Statistical analysis

Statistical analysis was conducted using SPSS version 22 (SPSS Inc., IL, USA). Descriptive statistics included the mean and standard deviations to assess. A Uni-variate linear regression was performed to assess linear dependence between Insertion torque and Predictive factors. Correlation was judged very strong from 1 to 0.9, strong from 0.9 to 0.7, moderate from 0.7 to 0.5, low from 0.5 to 0.3 and poor from 0.3 to 0. The alpha risk was set to 0.05. A multivariate linear regression was performed to assess the relation between Insertion torque and the explanatory variables: Imaginary pixel unit (IPU), Sinus Floor Thickness (SFT), Crestal cortical Thickness (CCT), Bone diameter (BD), and Initial bone height (IBH). Data were checked for multicollinearity with the Belsley-Kuh-Welsch technique. Heteroskedasticity and normality of residuals were assessed respectively by the Breusch-Pagan test and the Shapiro-Wilk test. A *p*-value 0.05 was considered statistically significant. The Newey West correction for heteroskedasticity was applied.

## Results

### Patient characteristics

This retrospective study was done on 113 patients (53 males, 60 females) with an averaged age of 48.6 years (range, 28–59 years).

### Clinical and radiographic analysis

A total of 124 transalveolar sinus lift procedures with simultaneous dental implant placement were done. The mean insertion torque was 28.38 N.cm (range, 20–43 N.cm). The initial bone height (IBH) ranged from 5.1 to 7.8 mm with mean 6.36 ± 0.89 mm. The crestal thickness (CT) ranged from 0.2 to 1.2 mm with mean 0.63 ± 0.28 mm. The sinus floor thickness (SFT) ranged from 0.2 to 1.3 mm with mean 0.75 ± 0.28 mm. The imaginary pixel unit (IPU) ranged from 112 to 443 HU with mean 112 ± 102.68 HU. The bone diameter (BD) ranged from 5.7 to 13.2 mm with mean 9.16 ± 165 mm. (Table 1). The variables studied according to the implant location are shown in Table 2.

**Table 1:** The descriptive statistics of variables.

	IT	IBH	ART	CCT	SFT	BD
Mean	28.38	6.36	9.16	0.63	0.75	289
SD	6.29	0.89	1.65	0.28	0.28	102
Median	27	6.2	8.9	0.7	0.7	314
Max	43	7.8	3.3	1.2	1.3	434
Min	20	5.1	0.8	0.2	0.2	112

**Table 2:** The Variables according to implant position.

Position/ Variable	First Premolar	Second Premolar	First Molar	Second Molar
**N**	8 (6.5%)	35 (28.2%)	39 (31.5%)	42 (33.8)
	mean ± SD	mean ± SD	mean ± SD	mean ± SD
**IT**	35.5 ± 5.8	30.1 ± 5.6	27.6 ± 6.7	26.2 ± 4.8
**IBH**	6.95 ± 0.4	6.4 ± 0.8	6.3 ± 0.95	6.3 ± 0.88
**CCT**	0.6 ± 0.3	0.6 ± 0.9	0.65 ± 0.2	0.6 ± 0.2
**SFT**	0.65 ± 0.2	0.8 ± 0.2	0.74 ± 0.3	0.7 ± 0.2
**IPU**	395.6 ± 36.4	326.4 ± 74.3	257.3 ± 105	269.2 ± 105.3
**BD**	8.9 ± 1.1	9.1 ± 1.7	9.3 ± 1.6	9.03 ± 1.62

### Uni-variate analysis

In Uni-variate analysis, Spearman’s correlation was used to assess linear dependence between Insertion torque and Predictive factors. Spearman’s correlation analysis showed (Table 3):

- A strong positive correlation was found between Insertion torque and Imaginary pixel unit.
- A low positive correlation was found between Insertion torque and Sinus Floor Thickness and between Insertion torque and Crestal Cortical Thickness.
- No correlation was found between Insertion torque and IBH, and between IT and BD.

**Table 3:** Uni-variate analysis.

	IT	IBH	BD	CCT	SFT	IPU
Mean	28.73	6.36	9.16	0.63	0.75	289
SD	6.29	0.89	1.65	0.28	0.28	102
Spearman’s correlation		0.044	-0.071	0.39	0.44	0.79
*P*-Value		0.629	0.436	0.001	0.001	0.001
Result		No correlation	No correlation	low positive correlation	low positive correlation	strong positive correlation

### Multivariate analysis

In multivariate analysis (Table 4):

- IPU and CCT were associated with higher values of IT.
- IBH, BD, and SFT were not associated with the value of Insertion torque.

**Table 4:** Multivariate analysis.

variable	odds ratio	*p*-value
Intercept	13.39 [7.36;19.42]	2.42e−05
IBH	−0.352 [−0.949;0.245]	0.245
CCT	7.42 [4.76;10.09]	2.05e−07 (<0.001)
SFT	2.14 [−0.174;4.46]	0.0695
IPU	0.0422 [0.0376;0.0468]	4.73e−36 (<0.0001)
BD	−0.142 [−0.545;0.261]	0.487

According to the multivariate analysis, the generalized estimation equation can be formulated as follows:

$${{{\rm{IT}}}} = \,	13.39+(0.0422{{{\rm{\times }}}}{{{\rm{IPU}}}})+(7.42 \times {{{\rm{CCT}}}})+(-0.352 \times {{{\rm{IBH}}}}) \\ \quad	+(2.14 \times {{{\rm{SFT}}}})+(-0.142 \times {{{\rm{BD}}}})$$

## Discussion

This retrospective study involved 113 patients who underwent 124 transalveolar sinus lift procedures utilizing threaded bone expanders alongside dental implant placement without bone graft materials. The study aimed to explore the influence of bone-related factors on insertion torque values during transalveolar sinus lift procedures by standardizing the surgical technique and implant system across all patients. The study findings revealed a direct correlation between imaginary pixel unit (IPU) and crestal cortical thickness (CCT) with insertion torque values. Conversely, there was an inverse relationship with implant protrusion length (IPL). However, no significant correlation was observed between sinus floor thickness (SFT) and initial bone height (IBH) and insertion torque values.

Since its introduction by Summer in 1994, the transalveolar sinus lift approach has become essential for posterior maxilla rehabilitation [28, 29]. This approach requires adequate bone height to support dental implants while minimizing elevation of the Schneiderian membrane [29]. When bone height is less than 5 mm, direct intervention on the Schneiderian membrane is necessary to prevent perforation [30]. Transalveolar sinus lift techniques can be categorized into two main types based on their underlying principles [31]: The first category involves fracturing the maxillary sinus floor and displacing it apically along with the attached Schneiderian membrane, as seen in osteotomies and expander techniques. In contrast, the second category entails delicately removing the sinus floor and apically displacing the Schneiderian membrane using specialized drills or through static or dynamic hydrostatic pressure [31].

The threaded bone expanders utilized in this study operate on a principle akin to the osteotome summers technique, advancing gradually and gently through the implant site to fracture the sinus floor and push it apically [13, 14, 27, 32]. Threaded bone expanders offer an advantage over the osteotome summer’s technique by providing greater comfort for the patient as they eliminate the need for a hammer [13, 14]. The utilization of threaded bone expanders in transalveolar sinus lift procedures has been well documented in medical literature, highlighting high success rates and significant bone gain [13, 14, 27, 32, 33]. The posterior maxilla is characterized by low bone density, necessitating special techniques to enhance primary stability, such as the under-preparation technique and bone spreading technique [34]. The threaded bone expanders employed in this study provide a controlled lateral bone condensing, resulting in heightened imaginary pixel unit in the area and enhancing implant insertion torque [13, 14, 27].

Various factors influence primary stability, including the surgical technique [35, 36] (drill diameter-implant diameter relationship) and the macroscopic morphology of the implant [37] (design, length, diameter, thread depth, and pitch). To ensure consistency, this study standardized both the surgical technique (utilizing threaded bone expanders) and the implant system (INNO submerged; Cowellmedi Inc, South Korea). While primary stability is important, it serves as a stepping-stone towards the ultimate goal of implant osseointegration. There is no universally agreed-upon minimum insertion torque needed for achieving osseointegration. However, oral surgeons generally consider an insertion torque within the range of 20 to 40 N.cm as indicative of “ideal” primary stability during implant placement [38, 39]. In many cases, high insertion torque ( > 50 N.cm) can be counterproductive as demonstrated by several clinical studies. It has been observed to potentially induce peri-implant bone necrosis by compromising blood supply, resulting in bone resorption around the implant, and ultimately increasing the risk of implant failure [40].

The hybrid surface macro-design implants, which utilized in this study, feature a distinctive design that serves two crucial purposes. Firstly, the cylindrical coronal portion of the implant ensures the attainment of sufficient primary stability. Secondly, the tapered apical portion of the implant aids in insertion, promoting ease of implant placement while maintaining proper alignment [41]. This relationship elucidates our findings that the thickness of the crestal cortical bone correlates directly with the insertion torque values. This also clarifies that sinus floor thickness does not affect insertion torque values. This is because the tapered apical portion of the implant, which interfaces with the cortical bone of the sinus floor, does not significantly contribute to achieving primary stability [42].

Hsu [42] noted that bicortical fixation in the posterior maxilla, where the implant engages both the crestal bone and the sinus floor, did not lead to higher primary stability values. The author attributed this to the use of hybrid implants, where the diameter of the apical portion of the implant is smaller than the diameter of the prepared implant site. An argument can be made that with the use of hybrid implants, the cortical bone of the sinus floor may not contribute significantly to enhancing primary stability.

Numerous clinical studies and systematic reviews [43–45] have highlighted a positive association between bone quality and primary stability when implants are inserted into fully vital bone. To provide clarity, bone quality, as defined by the majority of authors who have developed bone quality classifications [43, 45], is typically attributed to two primary components: crestal cortical thickness and imaginary pixel unit. Our study aligns with this notion, as the segment of the implant crucial for primary stability (the coronal portion) in transalveolar sinus lift procedures remains situated within fully vital bone.

Various surgical techniques have been suggested for implant preparation to enhance primary stability. These methods encompass under-preparation, osseodensification, osteotomies, bioactive kinetic screw (BKS) technique, and bone spreading through threaded bone expanders. In their investigation using fresh human cadavers, Pommer et al. [46]. discovered a notably significant positive correlation between radiographic bone density and insertion torque when placing tapered dental implants following socket preparation with the under-preparation technique. They observed that residual bone height had no impact on insertion torque values, aligning with the findings of our own study. In another study conducted by Rues et al. [47], where the under-preparation technique was employed to implant cylindrical dental implants in animal models, it was noted that residual bone height did not influence insertion torque. Instead, the key factors influencing insertion torque were bone density and the overall thickness of cortical bone. In this study, the authors utilized the term ‘total cortical thickness’ without specifically addressing the impact of the upper layer (crestal cortical thickness) or the lower layer (floor thickness) on insertion torque. We posit that this differentiation may not be crucial, given the cylindrical shape of the implants utilized. It can be inferred that sinus floor cortical thickness influences insertion torque for cylindrical implants but not for tapered or hybrid implants.

The limitations of the study include 1. The use of a singular technique for preparing the implant bed (threaded bone expanders) without comparing it to alternative techniques. Since insertion torque is greatly influenced by the surgical method, altering the technique for preparing the implant bed may lead to significantly different outcomes; 2- Employing a particular type of implant shape (hybrid implants), limiting the generalizability of results to conical or cylindrical implants; 3. The study relies solely on the insertion torque method for evaluating primary stability, without considering other assessment methods, such as resonance frequency analysis. This alternative method provides a more accurate measurement of bone-to-implant contact (BIC), while insertion torque can be significantly influenced by the amount of cortical bone present.

## Conclusions

In light of the limitations of our study, we conclude that the insertion torque values in transalveolar sinus lift procedures using threaded bone expanders, alongside the placement of hybrid implants (cylindrical in the coronal section and conical in the apical section), are primarily influenced by two factors: cortical bone thickness and imaginary pixel unit. However, it is important to acknowledge that the exclusive use of this method for evaluating primary stability and the focus on a specific implant shape may restrict the generalizability of our findings. Additionally, while sinus floor thickness, initial bone height, and bone diameter did not appear to affect insertion torque values in our study, further research utilizing diverse surgical techniques and implant types is necessary to comprehensively understand the relationships among these variables. Thus, our conclusions should be interpreted with caution, considering the potential impact of these limitations on the overall results and their applicability.

## Supplementary information

Flow Chart

## Data Availability

The data that support the findings of this study are available from the corresponding author, upon reasonable request.
