# Supplementary material for: Predictive factors for insertion torque values in transalveolar sinus lift procedures
Source: BDJ Open. 2025 Mar 3;11:23. doi: 10.1038/s41405-025-00297-w (PMC11876670; doi:10.1038/s41405-025-00297-w)
Supplement: Supplementary file 1 — Flow Chart [file 41405_2025_297_MOESM1_ESM.docx]

Excluded (n=18)

TSFE procedures included immediate implant placement (n=8)

TSFE procedures included using of any bone graft or bone substitutes materials (n=6)

TSFE procedures included bone expansion (n=2)

Previous maxillary sinus surgery (n=2).

Finally enrolled

(n=113)

Excluded (n=48)

(They did not meet the inclusion criteria)

No CBCT available before surgical procedure (n=14)

TSFE procedures were performed using other methods (n=23).

IBH < 5 or IBH > 8 (n=11)

Assessed for enrollment

(n=179)

Fulfilled inclusion criteria

(n=131)
